# The role of the residence-effect on the outcome of intergroup encounters in Verreaux’s sifakas

**DOI:** 10.1038/srep28457

**Published:** 2016-06-22

**Authors:** Flávia Koch, Johannes Signer, Peter M. Kappeler, Claudia Fichtel

**Affiliations:** 1 Behavioral Ecology and Sociobiology Unit, German Primate Center, Göttingen, Germany; 2 Department of Wildlife Science, University of Göttingen, Göttingen, Germany; 3 Department of Sociobiology and Anthropology, University of Göttingen, Göttingen, Germany

## Abstract

Intergroup competition has an important impact on the survival and fitness of individuals in group-living species. However, factors influencing the probability of winning an encounter are not fully understood. We studied the influence of numerical advantage and location of the encounter on the chances of winning in eight neighboring groups of Verreaux’s sifakas (*Propithecus verreauxi*), in Kirindy Forest, western Madagascar. Intergroup encounters were inferred from spatial data collected via GPS loggers over a period of two years. Location, i.e., the proximity to the respective core area, rather than the numerical advantage of a group in a given encounter, influenced the probability of winning. Accordingly, the high value that resident groups attribute to exclusive and intensively used areas increased their motivation in defending these locations against intruders. Moreover, losers used the encounter area less often than winners within a month after the encounter, suggesting that losing also entails long-term costs. Thus, our results suggest that in gregarious animals the particular circumstances of each encounter, such as the location, can outweigh group characteristics and predict the chances of winning an intergroup encounter.

Intergroup competition is a crucial aspect in the life of group-living animals because it mediates access to important resources such as food and/or mating opportunities. Therefore, it has an important impact on the survival and fitness of individuals. In addition, direct competition during group encounters can involve high levels of aggression, which has potential fitness implications for all individuals in the group^1^. After a decided encounter, winners will enjoy the benefits of accessing the contested resource, whereas losers will suffer the costs of defeat^2^,^3^. Potential costs of losing an intergroup encounter can range from alteration of travelling parameters (distance, speed, and sinuosity), increasing energetic demands for losers^2^, to the permanent loss of access to resources, which can lead to group dissolution in the long-term^4^,^5^.

Disputes over access to resources are often decided based on asymmetries between the contestants^6^. These asymmetries have been described as the “*Pay-off asymmetry”* and the *“Asymmetry in fighting ability*”^6^. The first refers to differences among individuals in their interest in defending resources, while the second is related to differences in the power in defending resources, known as the *resource holding power* (RHP)^6^. In group-living animals ranging from insects to primates, encounters tend to be decided based on asymmetries between contestants that can be present on the individual level, with stronger individuals tending to defeat weaker ones, but also on the group level, with an effect of numerical advantage, where large groups defeat smaller ones^3^,^7^,^8^,^9^,^10^,^11^,^12^,^13^,^14^,^15^,^16^,^17^,^18^,^19^. Numerical advantage is therefore an important general predictor of the outcome of intergroup encounters.

Since resources are rarely uniformly distributed, the economic value of areas varies with the availability of resources. Accordingly, animals should show variable levels of motivation in defending different areas within their home range^10^,^11^,^17^,^20^,^21^. Areas of intensive use can have a high value because, among other factors, residents are familiar with the distribution and availability of resources in this part of the home range^21^. This phenomenon, called the *residence effect,* suggests that due to differences in the economic value attributed to the area, residents have higher motivation to defend the area and as a consequence, higher chances of winning encounters than intruders^21^. In fact, in several species the location of encounters seems to be a better predictor for the outcome of a conflict than the numerical advantage between groups^11^,^17^,^20^,^22^. However, it is still not well known how these two factors or their interaction determine the outcome of group encounters.

We set out to study the influence of asymmetries in RHP, i.e. differences in group size and location of group encounters on the outcome of intergroup conflicts in Verreaux’s sifakas (*Propithecus verreauxi*), a group-living primate from Madagascar. Verreaux’s sifakas are a suitable and interesting species to investigate these questions because they live in relatively small groups of about 6 ± 2 individuals, but the largest groups can be up to five times bigger than the smallest ones^23^,^24^,^25^. Additionally, they exhibit an interesting pattern of territorial behavior, characterized by partial home range overlap and core areas for exclusive use^23^,^26^. In a field study on eight neighboring groups of Verreaux’s sifakas, we tested the influence of numerical advantage and the location of encounters on the outcome of group encounters. Moreover, we investigated the costs of losing group encounters related to alteration in traveling patterns and access to encounter areas after defeat.

## Methods

### Study site and species

The study was conducted from March 2012 to May 2014 in Kirindy Forest, western Madagascar (44°39′E, 20°03′S). We inferred intergroup encounters among eight neighboring groups from spatial data obtained from GPS loggers. One individual per group was equipped with a GPS logger (E-OBS Digital Telemetry GmbH, Gruenwald, Germany), recording locations continuously over a period of 3–4 months every 15 min from 4:30 h to 20:30 h (Table 1); the time settings were chosen based on the diurnal pattern of activities of sifakas^27^. We equipped subjects with GPS collars during brief anesthesia after blow-pipe darting^28^. Anesthetized individuals recovered within two hours and were returned to their social groups. Since sifakas are cohesive in their movements^29^, one GPS logger per group was sufficient to infer movements and the occurrence of intergroup encounters. In addition, we could include group size in the analyses because during directly observed encounters between the study groups all members were present. Group size in the study population varied during the course of the study period between three to eight individuals.

### Group encounters

The distance between groups used to infer intergroup encounters from the spatial data was based on the direct observation of 71 encounters in the field. We first calculated the mean duration of the observed encounters (23 ± 22 min). Next, we tested different possible encounter distances ranging from 15 to 150 m. For each proposed distance, we calculated the duration of encounters that would have occurred by counting the number of GPS relocations that were separated by the proposed encounter distance or less. Finally, we selected the encounter distance that resulted from the smallest difference (in absolute terms) between the calculated and the observed average encounter duration, which was 42 m (Supplementary Fig. S1). We therefore defined the beginning of an encounter when two groups were 42 m or less apart from each other, and the end when they were again at a distance of more than 42 m from each other for more than one hour. We set one hour as a conservative time limit based on the average duration of observed encounters. We defined the winner of the encounter as the group that stayed in the encounter area, i.e., the group that had the higher number of relocations (GPS points) within the encounter area within the hour following the end of the encounter. The loser of the encounter was defined as the group that left the encounter area. To analyze the data we randomly assigned the groups as being either a focal group or an opponent group.

For each encounter, we mapped an encounter area, which was defined as a buffer around all relocations during an encounter for both participating groups. The buffer width was set as the encounter distance 42 m + 1 m. Additionally we estimated kernel density home ranges (reference bandwidth^30^) with relocations that were recorded one month prior to the encounter, using R (CRAN) and package rhr (version 1.2.905)^31^. We then calculated the overlap between core areas (50% isopleth) and the encounter area.

### Location

We estimated total home range size (95% isopleth), core areas (50% isopleth) with kernel density home ranges (reference bandwidth^30^) based on relocations that were recorded one month prior to the encounter using the package rhr in R (version 3.1.2)^32^. Kernel density estimation is a non-parametrical statistical method for obtaining probability density surfaces from telemetry data, and it is commonly used to investigate ranging patterns of wild animals^33^,^34^. To examine whether the location of an encounter had an effect on its outcome we examined two aspects: first, we calculated the relative proportion of overlap between the encounter area and the core area of each group (proportion of overlap of focal group minus proportion of overlap of opponent group) during the month before the encounter, which we called *proportion of overlap*. Therefore, this measurement referred to the relative proportion of GPS relocations of the encounter area that were within the core area of each group. Accordingly, a higher *proportion of overlap* of one group reflects a higher overlap between the encounter area and the core area compared to their opponent. Since sifakas changed the location of their core areas (intensively and exclusively used areas) within their home ranges, each 2–4 weeks (Supplementary Fig. S2), we used the relocations from a month before the encounter to calculate the core area. Second, we calculated the distance between the encounter and the center of the home range of each group. For that we calculated the distance between the first encounter point and the centroid of the home range of each group based on the entire dataset, since the location of the center of the home range was stable over the study period (Supplementary Fig. S3).

### Costs of defeat

To infer short-term costs of defeat we compared travelling parameters (distance, speed, sinuosity) between winners and losers from each group within 1, 2, and 3 h after an encounter. The distance travelled was inferred through the sum of consecutive step lengths within 1, 2 or 3 h after the encounter; speed was calculated by dividing distance by time; and sinuosity by dividing distance by the straight line distance between the first and last relocation within the time frame. To assess long-term costs of losing an encounter, we compared the intensity of use (relative density of relocations) of the encounter area between winners and losers from each group for one month following an encounter, which we called the *post-encounter effect*. The relative density of relocations was based on the fraction of relocations in the encounter area within the month after the conflict.

### Statistical analyses

We used a binomial test to investigate whether one group of a given dyad won encounters more often than expected by chance. Binomial Generalized Linear Mixed Models (GLMM) from the package lmer4^35^ R version 3.1.2 were used to investigate the predictors for outcome of group encounters. We tested two different measurements for the location of an encounter: the *proportion of overlap* and the distance from the encounter area to the center of the home range of each group. In the first model, we included relative group size (group size of the focal group minus group size of the opponent group) to estimate the numerical advantage, and the *proportion of overlap* between the encounter area and the core area of each group involved in the encounter (arcsine square root transformed). In the second model, we included the relative group size and the distance from the encounter area to the center of the home range of each group (Table 2). In both models dyadic identity of the groups involved in the encounter was included as random factor. We controlled for interaction effects between predictor variables in both models but did not report them because they were not significant. We used maximum likelihood ratio tests to verify whether fixed factors explained a significant amount of the variance and to test the final model with fixed factors against the null model including only the random factors^36^. Wilcoxon signed-rank tests were used to investigate potential costs of losing an encounter (Table 2). Comparisons of potential costs of losing an encounter after 1, 2 and 3 h after the encounters were Bonferroni corrected, resulting in a p-value of 0.02 for these comparisons. All statistical analyses were performed in R version 3.1.2^32^.

### Ethical approval and informed consent

This study is in accordance with the German and Malagasy (Commission Tripartite CAFF) legal and ethical requirements of appropriate animal procedures. Consultation of the Animal Welfare Body of the German Primate Center is documented (No. 4–15). Research protocols and capture procedures were approved by the Ministry for the Environment, Water and Forests of Madagascar (MINEEF).

## Results

**Figure 1:**
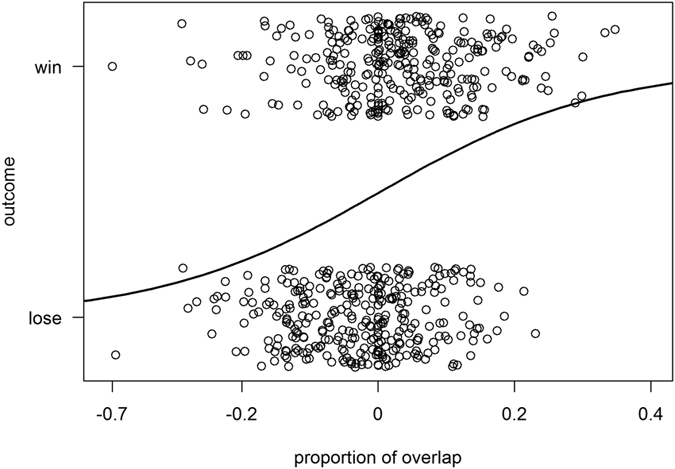
Effect of the *proportion of overlap* between the encounter area and the core area of focal and opponent group on the outcome of group encounters.

We recorded 759 encounters among eight neighboring groups, of which 624 were decided according to our definition of winning and losing. Sifakas did not exhibit clear and stable intergroup dominance relationships, because the frequency of winning encounters differed significantly only between 2 out of 13 dyads (Table 3). The probability of winning a group contest was not influenced by the relative group size of the opponents, indicating that the numerical advantage of larger over smaller groups did not influence the chances of winning an encounter (Table 4). Instead, the location of encounters, i.e. *proportion of overlap*, was crucial, since only the proportion of overlap of intensively used areas predicted the probability of winning an encounter (Table 4, Fig. 1). Moreover, the distance between the encounter area and the center of the home ranges of the groups involved in the encounter did not influence the probability of winning the intergroup encounter (Table 5).

**Figure 2:**
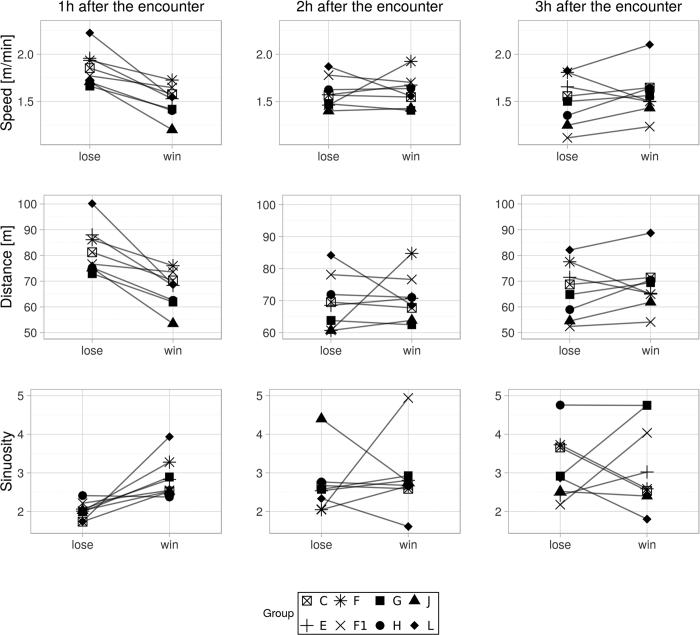
Short term costs in terms of alteration in travel parameters (speed, distance, and sinuosity) of each study group after winning and losing intergroup encounters in different time frames.

**Figure 3:**
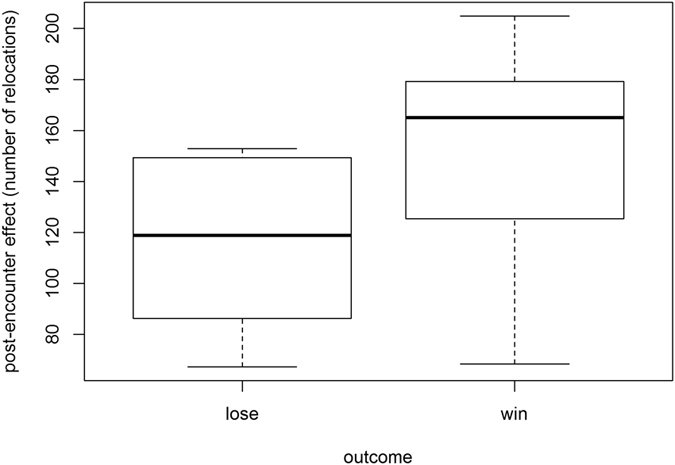
Long-term costs: *post-encounter effect*. Average of relative number of relocations per group in the encounter after winning and losing a group encounter.

The loser of an encounter travelled longer distances (Wilcoxon test: V = 36, p = 0.008), straighter (Wilcoxon test: V = 1, p = 0.01), and with higher speed (Wilcoxon test: V = 36, p = 0.008) than the winner (Fig. 2), but only within the first hour after the encounter (Fig. 2). Losers used the encounter area less often than winners within a month after the encounter, suggesting that losing also entails long-term costs (Wilcoxon test: V = 0, p = 0.007, Fig. 3).

## Discussion

Our results revealed that sifakas do not exhibit clear intergroup dominance relationships because in the majority of dyads no group consistently dominated an opponent group. The given numerical advantage did not influence the outcome of intergroup encounters. Instead, the location, the *proportion of overlap*, but not the distance to the respective home range center, was the main predictor for the probability of winning an encounter. Thus, the current resource value of an area mainly influenced sifakas’ motivation to fight, irrespective of the numerical disadvantage. Sifakas also did not pay heavily in terms of additional travelling costs after losing an encounter, but losers used the encounter area less intensively than winners within a month after the encounter. This post-encounter effect suggests that sifakas face long-term costs of losing a group encounter.

When the potential benefits of group encounters are not equally shared among all group members, some individuals may free-ride^37^. In sifakas, resources are not shared equally within groups. Dominant individuals of both sexes enjoy increased access to resources, and the presence of free-riders among subordinates is common^23^,^38^,^39^,^40^,^41^. Hence, the lack of a numerical advantage effect observed in this study might be due to collective action problems^41^. Therefore, a better predictor of the RHP of groups and their chances of winning is probably the number of actual participants in each encounter, rather than differences in total group size, consistent with what Crofoot and collaborators proposed^11^.

In several other species, the location of the encounter also influenced the outcome of encounters in favor of residents^3^,^11^,^17^,^20^. Indeed, residents are more familiar with the distribution and availability of resources in the disputed area, which creates an asymmetry in RHP between contestants in favor of the residents^21^. Additionally, due to the high attributed value to the disputed area, residents face higher costs from losing an encounter than intruders. They are therefore expected to be more motivated to defend the area and, hence, to free-ride less often than intruders^6^. In contrast, the high potential for free-riding in intruder groups may decrease their RHP^22^,^41^,^42^,^43^,^44^,^45^,^46^. In sifakas, the residence effect was the main predictor of winning an encounter, suggesting that resident groups were able to overcome other asymmetries in RHP, such as a numerical disadvantage.

Potential costs of losing an intergroup encounter have so far been rarely studied. In white-faced capuchins (*Cebus capucinus*), losers had higher travel costs than winners, and the underlying change in travel patterns was still present on the next day^2^. Sifakas also showed alteration in travelling parameters after losing an encounter; however, the effect was present only within the first hour after the encounter. In that first hour, losers travelled on average 15 m more than winners, a distance that is still within the general average hourly activity range (mean 71 ± 43 m). Thus, the variation in travelling parameters observed probably does not represent a major absolute cost and is most likely explained by the retreat of the losers to their core area.

Another potential cost of losing an encounter is the inability of using the resources within the encounter area after an encounter. For instance, in yellow baboons (*Papio cynocephalus*), losers used the encounter area less often than winners^3^. Sifakas also showed a post-encounter effect, with losers using the encounter area less often than winners in the month following an encounter. In contrast to other species that are highly motivated to defend space irrespective of the actual level of use, such as white-faced capuchins^11^, sifakas are highly motivated to defend intensively used areas, likely due the availability of specific food resources.

In conclusion, our results support the assumption that the particular circumstances of group encounters, such as the location of the conflict, are crucial predictors for the outcome of encounters^3^,^11^,^17^,^20^,^22^,^41^. Since the particular conditions of each encounter ought to impede the establishment of stable dominance relationships between groups, our data support the notion that the dilemma between cooperation and competition in gregarious animals occurs at the individual level^3^,^11^,^41^,^46^. Hence, future studies should investigate individual characteristics and detailed environmental conditions to achieve a better understanding of the dynamics in intergroup competition. Finally, the direct costs of losing conflicts are rarely taken into account in studies of intergroup encounters, and further research may also help to elucidate the potential fitness consequences of defeat.

## Additional Information

**How to cite this article**: Koch, F. *et al.* The role of the residence-effect on the outcome of intergroup encounters in Verreaux’s sifakas. *Sci. Rep.* **6**, 28457; doi: 10.1038/srep28457 (2016).

## Supplementary Material

Supplementary Information

## Figures and Tables

**Table 1:** Number of GPS locations (one location every 15 minutes) and days in which the groups were equipped with GPS loggers during the study period from March 2012 to May 2014.

Groups	Locations	Days with GPS logger
C	33060	551
E	29100	485
F	27120	452
F1	21360	356
G	30180	503
H	32160	536
J	31860	531
L	10740	179

**Table 2:** Variables tested in each model to investigate the predictors for outcome in group encounters and the short and long-term potential costs of losing an encounter and the respective statistical tests applied.

	Variables tested		Statistical test
Probability of winning (yes/no)	Model 1: Relative group size and *proportion of overlap*		*Binomial GLMM*
	Model 2: Relative group size and distance to the center of home ranges		
Potential costs of losing an encounter	***Short-term costs:1, 2, 3 hours after the encounter***	***Long-term costs: one month after the encounter***	*Wilcoxon signed-rank test*
	Distance Speed Sinuosity	*Post-encounter effect (relative number of relocations in the encounter area)*	

**Table 3:** Total number of recorded encounters between the 8 neighboring groups of sifakas, and the frequency of won encounters for each group.

ID group 1	ID group 2	Number of encounters	Frequency of winning group 1	Frequency of winning group 2	Binomial test p-value
C	E	148	64	84	0.118
C	F	17	9	8	1
C	G	51	16	35	**0.011**
C	H	1	0	1	NA
C	L	13	7	6	1
E	F	69	36	33	0.81
E	G	10	5	5	1
E	H	79	42	37	0.653
F	F1	82	27	55	**0.003**
G	H	15	10	5	0.302
G	J	59	23	36	0.118
G	L	4	1	3	NA
H	J	76	40	36	0.731

**Table 4:** Results of the binomial Generalized Linear Mixed Model on the proportion of overlap between the encounter area and the core area of the focal and opponent group and the numerical advantage on the probability of winning the encounter.

Fixed effects	Estimate	Std. Error	Z value	Pr(>|z|)
(Intercept)	−0.06	0.08	−0.72	0.47
Proportion of overlap	5.54	0.86	6.43	0.001***
Relative group size	0.04	0.05	0.92	0.35

Likelihood ratio test comparing the full model to a null model containing only the random effect: χ^2^ = 49.03, df = 2, p < 0.001.

**Table 5:** Results of the binomial Generalized Linear Mixed Model on the distance from the encounter area to the center of the home range of the groups involved and the numerical advantage on the probability of winning the encounter.

Fixed effects	Estimate	Std. Error	Z value	Pr(>|z|)
(Intercept)	9.20	3.15	2.93	0.003
Distance to the center	−0.26	0.17	−1.49	0.14
Relative group size	0.08	0.08	1.01	0.31

Likelihood ratio test comparing the full model to a null model containing only the random effect: χ^2^ = 2.77, df = 2, p = 0.25.
