# Supplementary material for: The role of the residence-effect on the outcome of intergroup encounters in Verreaux’s sifakas
Source: Sci Rep. 2016 Jun 22;6:28457. doi: 10.1038/srep28457 (PMC4916469; doi:10.1038/srep28457)
Supplement: Supplementary Information [file srep28457-s1.pdf]

Supplementary Information

The role of the residence-effect on the outcome of intergroup encounters in Verreaux's sifakas.

Flávia Koch*[1], Johannes Signer[2], Peter M. Kappeler[1, 3], and Claudia Fichtel[1]

1. Behavioral Ecology and Sociobiology Unit, German Primate Center, 37077 Göttingen, Germany biokoch@gmail.com
2. Department of Wildlife Science, University of Göttingen, Büsgenweg 3, Göttingen, 37077, Germany
3. Department of Sociobiology and Anthropology, University of Göttingen, 37077, Göttingen, Germany

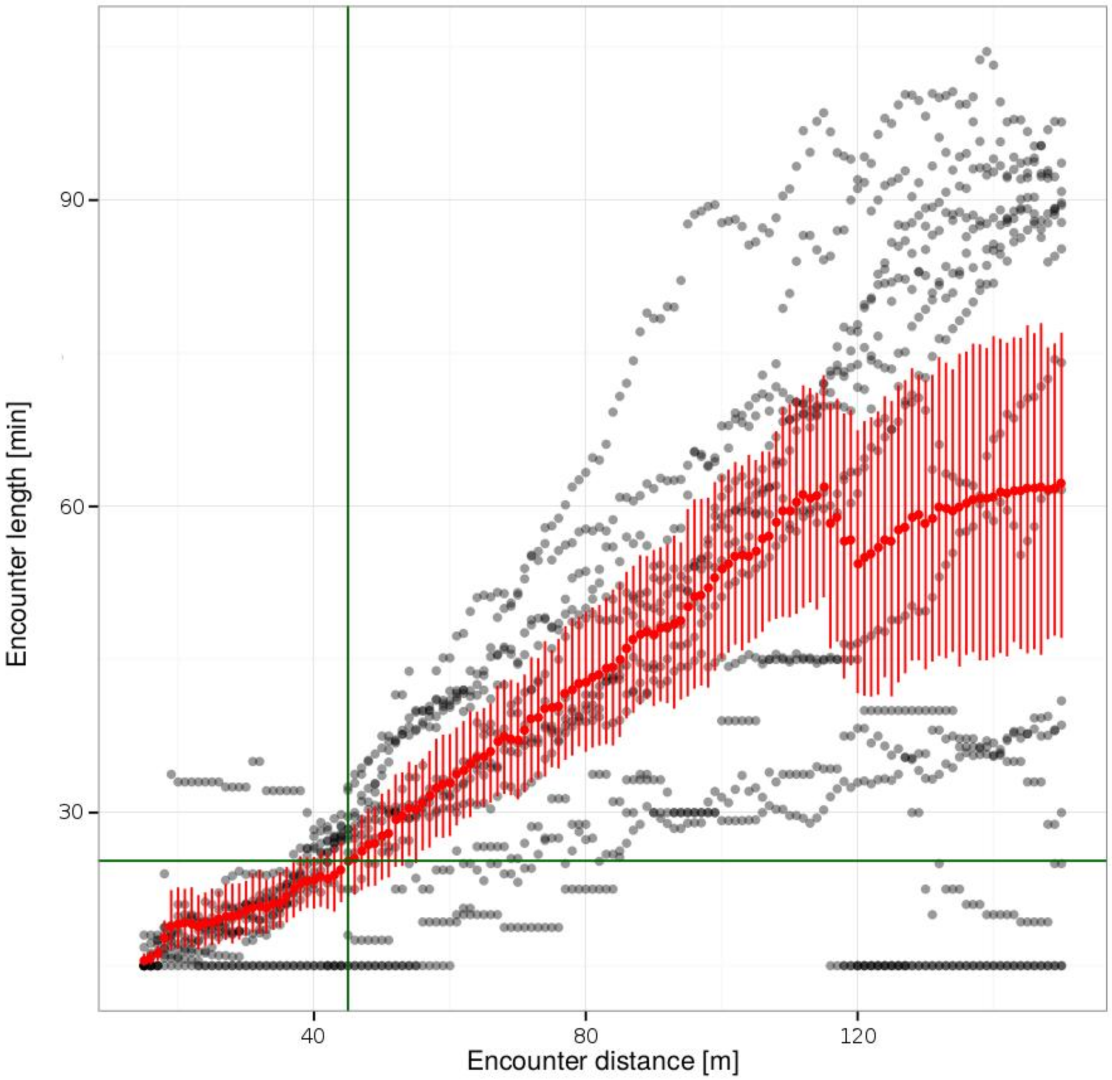

Figure S1. Estimation of distance between groups used to infer intergroup encounters from the spatial data. Different proposed encounter distances (x-axis) are plotted against the encounter duration from the corresponding proposed distance (y-axis). The horizontal green line indicates the empirically observed encounter duration, and the vertical green line marks the encounter distance at 42m. The selected encounter distance of 42m revealed the shortest difference (in absolute terms) between the empirically observed encounter duration and the encounter duration from proposed encounter distance.

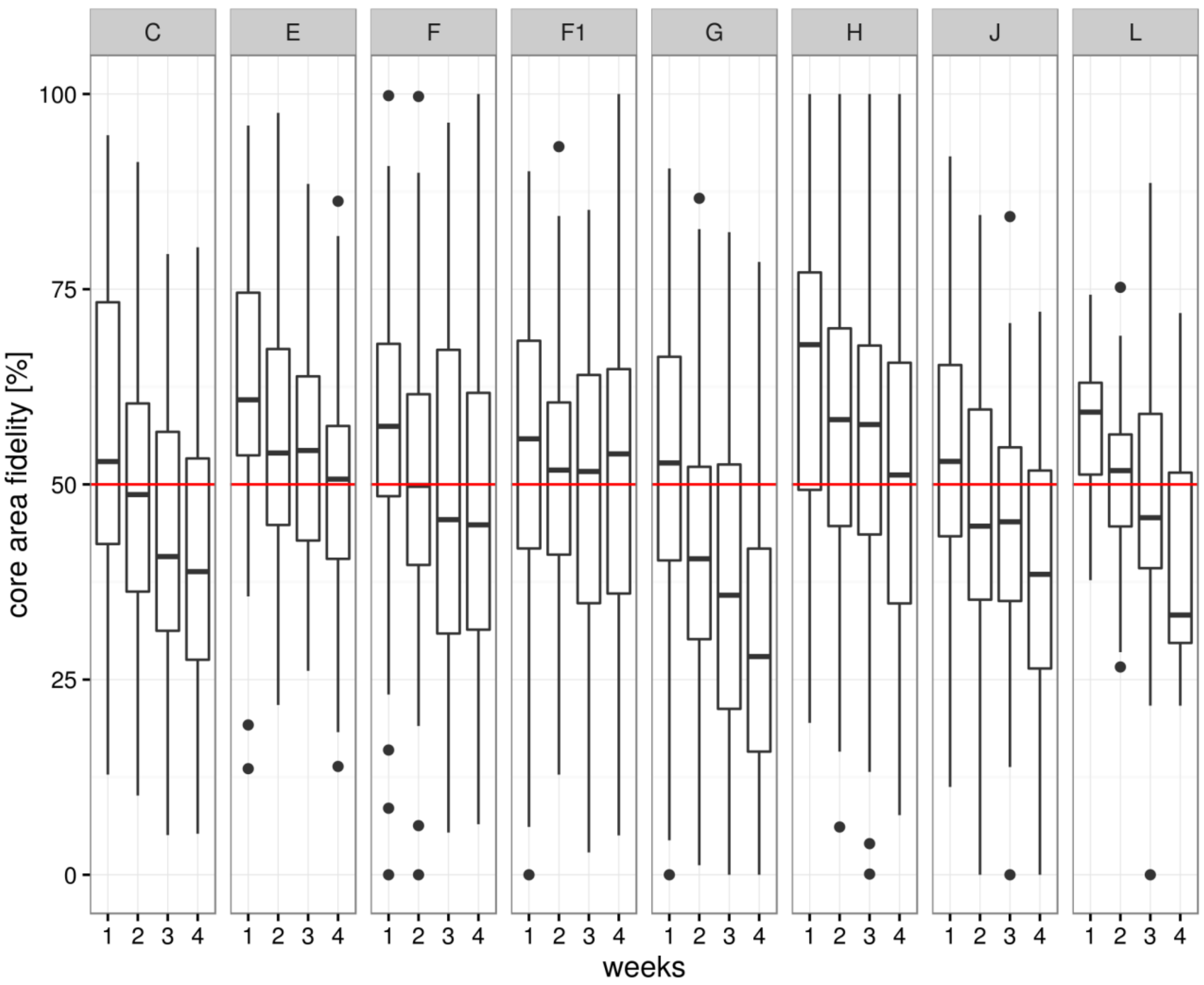

Figure S2. Weekly changes in the proportion of the overlap between the current and the former core area for each study group. Less than 50% of overlap means a switch of position of the current core area in relation to the position of the former one.

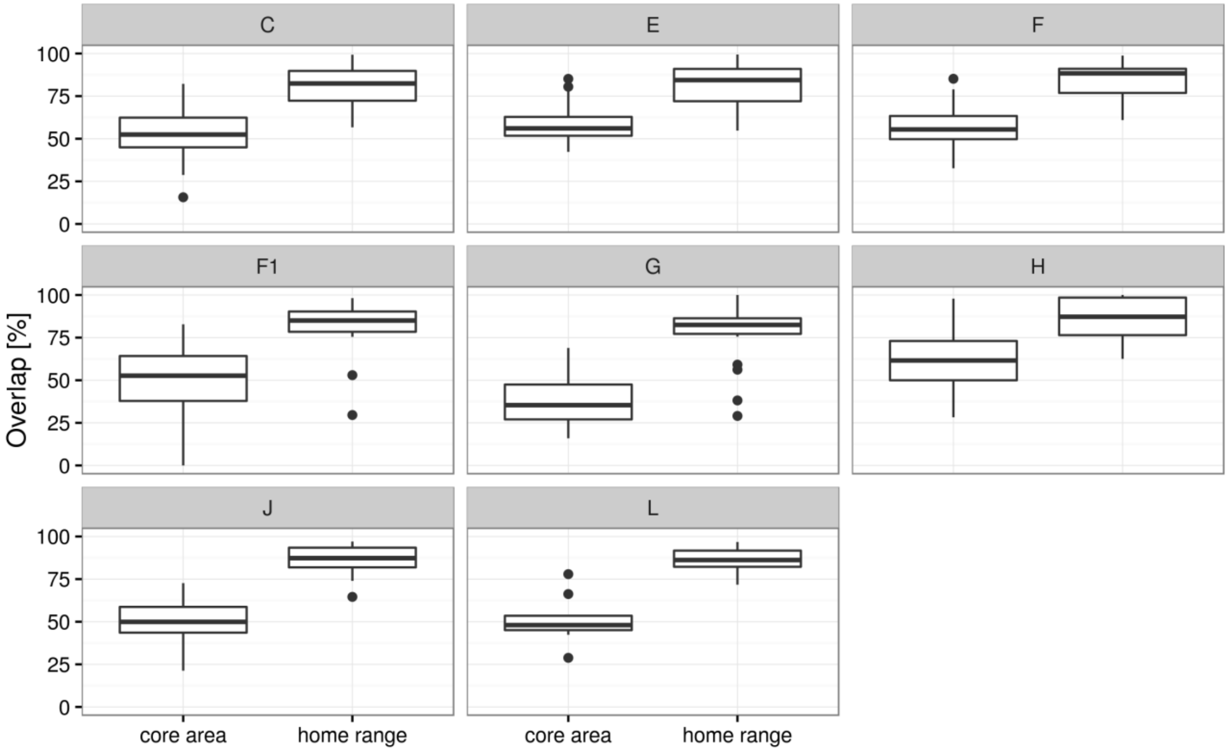

Figure S3. Stability of home range location across the study period based on kernel density home ranges. The y-axis shows the distribution of overlaps of monthly core areas (50% isopleth) and home ranges (95% isopleth). *Zero* means that the location of the home range changed completely, and 100% means that the location of the home range did not change.
